# Patients as Co‐Researchers in Oncology: A Qualitative Exploration of Participatory Governance

**DOI:** 10.1111/hex.70762

**Published:** 2026-07-09

**Authors:** Yaël Busnel, Aline Rollet, Aline Cristiani, Véronique Rivoire, Laurie Panse, Julie Haesebaert, PROOF Investigators Group, Kepenekian Vahan, Dalle Stéphane, Sébastien Couraud, Mellie Heinmann, Hallouche Nabil, Tournigand Christophe, Wassermann Johanna, Boissel Nicolas, Rabian Florence, Mebarki Soraya, Pauline Maisani, Aurélien Troisoeufs, Pascale Sontag, Magalie Girodet, Mathilde Lochmann, Véronique Christophe, Stéphane Cognon, Sarah Prudhomme, Claude Ganter, Alice Gros, Alexandra Villatte, Anne Termoz

**Affiliations:** ^1^ Research on Healthcare Performance RESHAPE, INSERM U1290 Université Claude Bernard Lyon 1 Lyon France; ^2^ Assistance Publique – Hôpitaux de Paris (AP‐HP) Paris France; ^3^ Hospices Civils de Lyon Lyon France; ^4^ Hospices Civils de Lyon, Pôle Santé Publique Unit Recherche et Epidémiologie Cliniques Lyon France; ^5^ Centre Léon Bérard Lyon France; ^6^ GHU Paris Psychiatrie &; Neurosciences Paris France

**Keywords:** governance, oncology, participatory action‐research, patient and public involvement, patient partners, peer support, qualitative study

## Abstract

**Background:**

Patient and public involvement (PPI) is increasingly promoted in health services research, yet empirical evidence remains limited on how participatory governance is operationalised in research practices.

**Methods:**

This qualitative study explored patient partners' experiences of involvement in the PaRole OncO France (PROOF) research project, a multicentre participatory action research (PAR) project aimed at adapting, implementing and evaluating a peer support intervention in oncology. Semi‐structured interviews were conducted with patient partners involved in project governance. Data were analysed using inductive thematic analysis, with patient partners involved in validating analytic outputs.

**Results:**

Seven of the eight invited patient partners (PPs) were interviewed. Three interrelated themes emerged: (1) personal invitation as recognition: a catalyst of engagement, whereby being personally solicited was experienced as recognition of experiential legitimacy; (2) securing a ‘real place’: relational trust, dedicated spaces and institutional frictions, capturing both the conditions enabling genuine co‐governance (dedicated spaces, recognition of experiential knowledge and responsive research teams) and the administrative and temporal constraints that strained it; and (3) reciprocal transformation: perceived impacts on the research and on personal trajectories. Patient partners perceived meaningful contributions to intervention design and team cohesion, alongside personal benefits such as skills development and network building. Persistent barriers were primarily structural, including administrative constraints, research temporalities and regulatory procedures.

**Conclusion:**

PROOF illustrates how intentional participatory governance mechanisms can support sustained PPI in research. Addressing administrative barriers, clarifying roles and nurturing trust and communication are crucial for meaningful participation in oncology research.

**Patient or Public Contribution:**

The qualitative data presented in this manuscript were obtained through interviews with patient partners. Patient partners co‐designed governance structures, participated in all committees, co‐facilitated co‐construction workshops, and contributed to data interpretation and manuscript validation.

**Abbreviations**
APs: accompanying patients
BEE: bidirectional engagement and equity research framework
COPIL: steering committee (comité de pilotage)
COPAR: participatory committee (comité participatif)
PPs: patient partners
PAR: participatory action research
PCORI: Patient Centred Outcomes Research Institute
PPI: public and patient involvement
PROOF: The PaRole OncO France project
SPOR: strategy for patient oriented research

## 1 Introduction

Patient experience is recognised as one of the five key components to achieving high‐quality healthcare organisation [1, 2]. Over the past two decades, patient and public involvement (PPI) has increasingly been promoted as a cornerstone of health services research [3, 4]. PPI is commonly described as conducting research *with* or *by* patient and caregivers, rather than *to*, *about* or *for* them [5]. Beyond ethical considerations, patient involvement is now framed as a lever for transforming research practices through the integration of experiential knowledge into the production of evidence [6].

This evolution is closely aligned with traditions of participatory action research (PAR) in public health. The PAR approach is one based on core principles of participation of relevant stakeholders and embeddedness within the communities most affected by the services involved and the changes to them, rather than a set research design [7, 8].

Within this paradigm, patients are actively involved in defining priorities, shaping interventions, and contributing to decision‐making processes [9]. National and international initiatives such as INVOLVE in the United Kingdom, the Patient‐Centred Outcomes Research Institute (PCORI) in the United States, and the Canadian Strategy for Patient‐Oriented Research (SPOR) have contributed to institutionalising these principles and encouraging PPI across multiple stages of research [10, 11, 12].

Despite this growing policy and methodological consensus, translating participatory principles into practice remains challenging. Research attention has progressively shifted from questioning why patients should be involved to examining how meaningful involvement can be operationalised. While the benefits of patient involvement are widely reported, including increased relevance and acceptability of research, there is limited empirical evidence on how participatory research is organised and governed in real‐world settings [13, 14, 15, 16]. National and international initiatives have begun to articulate governance‐oriented recommendations for PPI. In Canada, the SPOR emphasises formal governance structures, explicit role definitions, patient representation in decision‐making bodies, and dedicated resources across all stages of research [12]. Similarly, INVOLVE and related NHS guidance frame PPI as research conducted with or by patients, advocating for their integration as partners within project governance, supported by coordination mechanisms, shared decision‐making and appropriate recognition [10]. Key questions persist regarding the conditions under which patient partnership becomes meaningful and sustainable with an appropriate recognition methods, and how governance structures shape patients' experiences as partners rather than participants [15, 17, 18, 19]. Strengthening the evidence base on the value and processes of PPI is therefore essential to addressing persistent barriers to its implementation [20].

These issues are especially salient in public health research addressing complex interventions [9]. Peer support interventions in oncology illustrate this complexity: they involve experiential relationships between patients, integration within healthcare teams, and institutional arrangements related to recognition, training and funding. Research‐action approaches are particularly suited to studying and developing these interventions, as they allow for iterative co‐construction and adaptation grounded in practice [9, 21].

The PaRole OncO France (PROOF) initiative was designed as a multicentre PAR. Its aim is to transfer, adapt and evaluate the PAROLE‐Onco programme, first developed in Quebec, to the French health system [22]. The Quebec programme integrated accompanying patients (APs) into oncology care pathways. APs are individuals who have experienced cancer themselves and are trained to provide support to current patients by sharing experiential knowledge, fostering emotional reassurance, and helping them navigate the care pathway [23]. In PROOF, patient partners (PPs) take on dual roles: some act as APs, providing direct support to other patients, while others (sometimes the same individuals) take on a co‐researcher role, contributing to governance, methodological discussions, data analysis and dissemination.

This article seeks to address current gaps in the literature by describing the participatory governance structure of the PROOF research programme and by exploring PPs' perspectives on their involvement during the first phase of the project. By focusing on their lived experiences, this study aims to better understand how participatory governance is enacted in practice, what facilitates or constrains meaningful involvement, and how patient partnership can be sustained within a large, multicentre public health research‐action project.

## 2 Materials and Methods

### 2.1 The PROOF Programme

The PROOF project is a PAR conducted into 10 oncology units located in four French healthcare institutions (three university hospitals and one comprehensive cancer centre) [24]. Its main objective is to design, implement and evaluate a peer‐to‐peer support intervention for people living with cancer, grounded in a participatory governance model. The project was developed collaboratively by researchers, healthcare professionals, institutional partners, and PPs.

The project is structured in two phases. The first phase focused on establishing participatory governance, co‐designing the peer‐to‐peer support intervention and preparing its implementation, while the second phase will evaluate the implementation process and outcomes of the intervention [25]. The present article focuses on Phase 1 and examines the experiences of PPs involved in the governance of the project (See Appendix S1).

**Figure 1:**
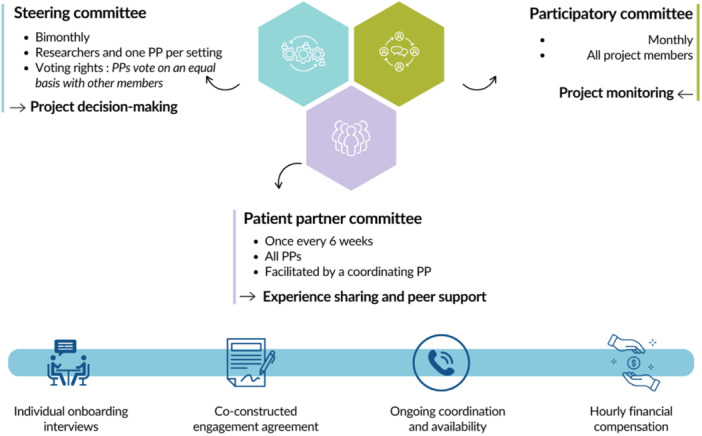
Participatory governance structure of the PROOF programme.

To ensure that PPs could contribute to the design, conduct and oversight of the research, four complementary participatory structures were established (Figure 1). Three were standing committees that included researchers and PPs and met at regular intervals throughout Phase 1; the fourth consisted of time‐limited co‐construction workshops held in each participating unit.

- *Steering committee (COPIL)*: bimonthly (1.5 h, online), including the coordinating investigator, project manager, doctoral researcher, the coordinating patient partner, and one patient partner per setting. The steering committee was the formal decision‐making body of the project: methodological, organisational, administrative and budgetary decisions were discussed and, when consensus could not be reached, put to a vote in which PPs held the same voting rights as the other members. The other structures did not hold formal decision‐making power; their input was systematically reported to the steering committee.

- *Participatory committee (COPAR)*: monthly (1.5 h, online), bringing together all project teams (researchers, healthcare professionals and managers, APs) and PPs to function as a cross‐site community of practice: a shared space in which members from the different units shared progress, identified common challenges, exchanged experiences and learned from one another across units.
- *Patient partner committee*: every 6 weeks (1.5 h, online), specifically dedicated to PPs, facilitated by the coordinating patient partner (L.P.) with support from a doctoral researcher (Y.B.). This space enabled peer exchange, mutual support, and contribution to study documents for drafting and revision.

*Co‐construction workshops*: unlike the three standing committees, the workshops were time‐limited working sessions organised in each participating oncology unit to co‐design the components of the peer‐to‐peer support intervention [25]. They brought together the unit's healthcare professionals, managers and APs, and were jointly facilitated by a member of the research team and a PP. Among the eight PPs, three (whose involvement centred on the partnership and research dimensions of the project) acted as co‐facilitators and contributed to writing the workshop reports, while the five PPs who were also current or future APs took part as participants, contributing their experiential perspective to the co‐design of the intervention. The design, conduct and outputs of these workshops are reported in detail elsewhere [25].

Within these structures, PPs could take part in a range of predefined missions across the PROOF programme, including the validation and development of study questionnaires, the coding and analysis of data, the writing of scientific articles, and the presentation of the project at conferences. The extent and form of each PP's involvement depended on the role(s) chosen in their individual engagement agreement and on their interests and skills: for instance, the coordinating patient partner contributed to the coding of qualitative data across several of the programme's studies. One contribution was not initially planned: the validation of the French‐language version of a questionnaire.

Beyond formal governance committees, specific relational and support mechanisms were implemented to facilitate and sustain PPs engagement throughout Phase 1 of the PROOF programme [24].

At the beginning of the study, each PPs participated in an individual meeting with a researcher (Y.B.) from the coordinating team. These meetings aimed to establish mutual understanding, explore patients' experiential backgrounds and motivations, present the objectives and organisation of the project, and co‐construct an individual engagement agreement. This engagement sheet detailed: (i) the role(s) chosen by the PPs (e.g., membership of one or more committees, co‐facilitation of co‐construction workshops, review, simplification or translation of study documents, contribution to dissemination activities such as conference presentations); (ii) the expected contributions and an estimated time commitment; and (iii) the practical modalities of involvement (preferred communication channels, availability, compensation arrangements). Roles were not assigned by the research team: each PPs selected their preferred forms of involvement according to their interests, skills and availability, and these choices could be revised over time during individual follow‐up meetings. The agreement was validated and signed jointly by the patient partner and the research team.

Throughout Phase 1, continuous support was ensured by a researcher from the coordinating team (Y.B.) and the coordinating patient partner (L.P.), who remained available to PPs via email or telephone and offered individual follow‐up meetings when needed. These contacts provided a space to address questions, clarify expectations, and discuss emerging difficulties related to participation.

PPs were financially compensated on an hourly basis for their involvement during Phase 1, at a rate of 43 € per hour, covering preparation and meeting time as well as travel expenses for conference. Payments were processed through the administrative services of each institution. This compensation reflected an explicit recognition of PPs' time and contribution and aimed to support more equitable conditions of participation.

### 2.2 Participants

The qualitative study included all eight PPs, all of whom had been actively involved in phase one of the PROOF research programme at different levels (Table 1) [27]. PPs had originally joined the PROOF programme through direct, personal invitation by healthcare professionals or researchers, each PP being attached to a single participating unit. Eligibility for the PPs role required having a personal experience of cancer and having completed active treatment (i.e., being in remission at the time of involvement); prior engagement in patient partnership, teaching or research activities was valued but not required. No criteria regarding specific training, age or cancer type were applied.

**Table 1:** Participatory governance committees and levels of patient partner involvement.^a^

Committee	Number of PPs	Missions	Level of involvement^a^
Coordination team	1	Coordinating patient partner committees Patient partner liaison Co‐creation of the workshop's framework Co‐creation of the APs interview guide	Coproduction
Steering committee	4	Decisions concerning project progress (methodology, administrative/financial issues) Writing articles Presentation at conferences	Coproduction
Participatory committee	8	Project progress on their unit Feedback on the workshops	Consultation
Patients' partners committee	8	Sharing and feedback on their integration into their respective units Peer advice Feedback on tools developed Document design	Consultation to Collaboration
Co‐construction workshops	4	Facilitating workshops Writing workshops reports	Coproduction
	4	Co‐construction peer support programme Sharing needs Feedbacks	Collaboration

^a^ In the Montreal model of patient partnership, patient involvement is conceptualised along a continuum of four increasing levels of engagement: (i) information (patients are kept informed about the research); (ii) consultation (patients' opinions are sought, without any obligation for the team to integrate them); (iii) collaboration (patients work alongside researchers on specific aspects of the project, with shared influence on the related decisions); and (iv) co‐production (patients and researchers share decision‐making power and responsibility throughout the process) [26].

The PPs identified during this phase were either APs already active in the institution (*n* = 3), or PPs already collaborating with the institution on other projects (*n* = 5); the latter group included individuals engaged in research or quality‐improvement activities as well as PPs preparing to take on an APs role (*n* = 2). These profiles also shaped the roles taken in the co‐construction workshops: the three PPs with a research/teaching background acted as co‐facilitators, whereas the five current or future APs participated as contributors (Table 1).

### 2.3 Data Collection

To explore and document the lived experiences of PPs involved in the project, semi‐structured individual interviews were conducted between May and July 2025, approximately 1 year and a half after the launch of the PROOF research programme. Interviews were conducted either online (*n* = 7) or in person (*n* = 1), depending on participants' preferences and availability. To contextualise PPs' involvement and document governance arrangements, we also collected relevant project materials produced during Phase 1, including meeting agendas and minutes from governance committees, workshop materials, and patient partner engagement documents (e.g., individual engagement sheets). These documents were used to describe the participatory structures and to support interpretation of interview data.

All interviews were carried out by an independent researcher (A.R.) who was not involved in the PROOF project, in order to reduce potential bias and facilitate open expression. An interview guide was developed based on PPI recommendations and internally tested by the research team (A.R., Y.B. and J.H.) (see Appendix S2). Interviews were audio‐recorded with participants' consent and transcribed verbatim. To ensure confidentiality, de‐identified summaries of interviews were produced by two researchers (A.R. and Y.B.).

### 2.4 Data Analysis

Qualitative data were analysed using an inductive thematic analysis, following Braun and Clarke's six‐phase approach (familiarisation with the data, generation of initial codes, construction of candidate themes, reviewing themes, defining and naming themes, and producing the report) [28]. Initial codes were generated inductively, at both semantic and latent levels, without using a pre‐existing coding frame. To strengthen analytical rigour, a double‐coding process was implemented. Two researchers (A.R. and Y.B.) independently coded a subset of transcripts and discussed discrepancies until consensus was reached. This process led to the development of a shared codebook, which was subsequently applied to all interviews using QDA Miner software (version 6.0.2). Themes were then developed by clustering codes into patterns of shared meaning across participants, reviewed against the coded extracts and the entire dataset, and iteratively refined and renamed. Although the interview guide addressed three broad domains (entry into the study, factors shaping participation, and perceived impacts; see Appendix S2), the themes were not predetermined by this structure: the analysis aimed to identify the patterns of meaning underlying participants' accounts within and across these domains (the centrality of personal invitation as a form of recognition, or the tension between feeling like an ‘alibi’ and having a ‘real place’).

During one virtual co‐analysis meetings, three PPs (L.P., V.C. and A.C.) and one researcher (Y.B.) reviewed and commented on the codebook and the table of verbatim extracts organised by theme [29, 30]. This step ensured that the analysis reflected not only academic interpretations but also the lived perspectives of PPs directly involved in the project. The bidirectional engagement and equity (BEE) research framework was not used during coding but was mobilised at the interpretation stage, in the Section Conclusion, 4, as a lens to organise the relationships between governance arrangements, engagement mechanisms and perceived impacts [31]. This framework was developed to guide community–academic partnerships, which conceptualises equitable engagement through two interdependent mechanisms, bidirectionality (the reciprocal exchange of knowledge, resources and influence between partners) and equity (the fair distribution of power, recognition and resources).

### 2.5 Ethical Considerations

The study received ethical approval by the Medical Ethical Review Board of the Hospices Civils de Lyon (2024‐01‐25‐04). All participants were informed about the objectives of the study, the voluntary nature of participation, and their right to withdraw at any time. Written informed consent was obtained for both participation and audio‐recording. Anonymity was preserved by using pseudonyms or initials in the presentation of results.

## 3 Results

### 3.1 Description and Characteristics of Respondents

A total of eight PPs were invited to interview and seven were interviewed; one PP could not be interviewed for scheduling reasons. Table 2 showed the sociodemographic characteristics of PPs.

**Table 2:** Sociodemographic characteristics of the participants (*N* = 7).

	Patient partner (*N* = 7)
	*N* (%)
Gender	
Female	6 (85.7)
Male	1 (14.3)
Age	
35–44 years old	2 (28.6)
45–54 years old	1 (14.3)
55–64 years old	4 (57.1)
Education level	
Associate's degree	2 (28.6)
Undergraduate or graduate degree	5 (71.4)
Time of involvement^a^	
Upon grant submission	4 (57.1)
Beginning of the study	2 (28.6)
During phase 1	1 (14.3)
Previous experience	
Had patient engagement training in research	1 (14.3)
Previous patient engagement experience in research	2 (28.6)

^a^ Time of involvement refers to the point at which each PPs joined the programme (mutually exclusive categories): ‘upon grant submission’ (involved from the development of the funding proposal, in 2023), ‘beginning of the study’ (joined at the official launch of the programme, in October 2023), or ‘during Phase 1’ (joined after the launch). All PPs remained involved from their entry point onward.

### 3.2 Implementation of Participatory Governance During Phase 1

Between October 2023 and October 2024, 12 participatory committee meetings (COPAR), 6 patient partner committee meetings, and 5 steering committee meetings (COPIL) were held. PPs attendance was documented for each meeting: COPAR meetings included 4–7 PPs per meeting (mean 5.5), the patient partner committee included 4–6 PPs (mean 5.0), and COPIL meetings included 1–4 PPs (mean 2.6), reflecting the different functions and membership rules of each governance space (Table 1).

Across Phase 1, PPs dedicated a total of 326 h to the project, with individual contributions ranging from 21 to 127 h; the upper bound corresponded to the coordinating PPs role. In addition to meeting participation, the patient partner committee contributed to tangible project deliverables, including the development of a workshop tool, the translation of a questionnaire into French, the review and refinement of study questionnaires, and the co‐development of implementation resources to support the next stage of the programme (APs' logbook and a practice charter for the community of practice). These outputs provide a documented record of patient partner contributions throughout Phase 1.

### 3.3 PPs Perceptions of Their Involvement in the PROOF Programme

Following inductive thematic analysis, three interrelated themes were identified: (1) personal invitation as recognition: a catalyst of engagement; (2) securing a “real place”: relational trust, dedicated spaces and institutional frictions; and (3) reciprocal transformation: perceived impacts on the research and on personal trajectories.

#### 3.3.1 Personal Invitation as Recognition: A Catalyst of Engagement

Beyond individual trajectories, the participatory and innovative nature of the PROOF programme itself emerged as the primary driver of engagement. Participants expressed interest in contributing to the adaptation of a model developed in Quebec and in engaging in a concrete PAR process: *Being able to evaluate the contribution of APs is very important… having tools is really great (PP06)*.

PPs described their initial engagement as largely triggered by direct solicitation from healthcare professionals or researchers involved in the project. Being personally invited was experienced as a strong signal of trust and recognition of their lived expertise and of the pathway they had built over time, which played a decisive role in their decision to participate. As one participant explained: *What motivated me was simply that the doctor asked me. She said she was part of a project and that it would be good to have patient partners. (PP04)*.

For several PPs, this invitation resonated with prior experiences of engagement in research, teaching, or peer support activities, which facilitated their entry into the project and reinforced their perceived legitimacy: *I was already involved in participatory projects (PP01)*.

However, early engagement was also characterised by initial tensions, particularly around legitimacy. Several participants described the research environment as intimidating: *Throughout the COPIL, you have to think about the right moment… what is my legitimacy as a patient partner compared to a researcher?* (PP01).

Participants emphasised that facilitation practices such as allowing silences, adjusting the meeting tempo and explicitly inviting patient partner input helped reduce intimidation and supported confidence over time.

#### 3.3.2 Securing a “Real Place”: Relational Trust, Dedicated Spaces and Institutional Frictions

The participatory approach is a fundamental component of PPs engagement. The recognition of experiential expertise facilitates a transition from a consultative approach to a genuine co‐construction model. This approach ensured that all parties involved were aligned with the overarching objective, particularly in the context of developing a roadmap from the ground up. This approach also fosters the development of a sense of belonging and legitimacy within the group. *First of all, even though I knew it, it was… extremely important in steering committees and project committees to include PPs and to learn, to see how to work with them, how to work on an equal footing with them so that they weren't just there as an alibi, so that they were heard, but so that they had a real place. (PP05)*.

For PPs involved in steering committees and workshop facilitation, this approach translated into a strong sense of full team membership. Roles were described as clearly defined from the outset and evolving within a climate of growing trust: *The role was unambiguously delineated from the commencement and functioned effectively throughout the duration of the workshops. (PP03)*.

The creation of dedicated spaces for PPs, particularly the patient partner committee, was identified as a key enabling condition. These spaces allowed PPs to express themselves freely, outside formal scientific committees, and facilitated exchanges between individuals from diverse settings. Over time, this contributed to the emergence of a shared identity: *It was fortunate that it existed and that it was disconnected. This allowed many of us to come together, even though our fields of practice were extremely different, but we found a lot of common ground on quite a few things. (PP05)*.

Participants also highlighted the responsiveness and adaptability of the research team as a crucial facilitator. Researchers' willingness to adjust organisational arrangements and integrate feedback reinforced trust and sustained engagement: *They [researchers] really listened to us. I know that adjustments were made and ultimately taken into account. (PP01)*.

Finally, acknowledging the diversity of PP profiles and creating a climate of respect and trust were seen as essential to maintaining involvement over time: *There was really, a respect, a trust that allowed us, as you said, to express ourselves in complete… in complete honesty, trust, and respect. (PP03)*.

### 3.4 Institutional Frictions: Administrative and Temporal Constraints

Despite overall positive experiences, participants identified significant barriers related to administrative and financial processes, particularly regarding remuneration. The diversity of employment and social statuses complicated payment procedures, leading to delays and frustration: *The only thing is that it really dragged on […] I know that there was a bit of frustration on that side. Whereas I know that it takes a long time to do, it's very complicated to find. (PP05)*. In practice, two successive delays affected remuneration. The first, upstream and the main source of the frustration reported by PPs, corresponded to setting up the payment circuit (validating the procedure and identifying the staff authorised to process payments) together with the individual administrative handling required by the diversity of PPs' employment and social statuses; this phase took approximately 7 months. The second, downstream and fixed, corresponded to the institutional payment cycle itself: once the procedure had been validated and the responsible staff identified, approximately 40 days were still required for the transfer to reach the PPs.

Some PPs nevertheless expressed understanding of institutional constraints, acknowledging the complexity of managing remuneration fairly within existing systems: *From experience, I know it takes a very long time. What's more, it's still a bit of a difficult problem to solve. (PP06)*.

Participants also pointed to tensions between the temporalities of research and their expectations. Regulatory and ethical procedures were perceived as slow and burdensome, generating impatience and, at times, disengagement: *There's a lot of red tape, a lot of administrative burdens that slow things down and we're not all working on the same schedule. (PP01)*.

For some, the delay in moving from the 1st codesign phase to the 2nd implementation phase was a source of frustration: *What exasperates me is the slowness of certain procedures. (PP07)*.

### 3.5 Reciprocal Transformation: Perceived Impacts on the Research and on Personal Trajectories

PPs described multiple, interrelated benefits associated with their involvement, affecting both the project and their own trajectories.

At the project level, participants perceived their experiential knowledge as enriching research processes and outputs. Their contributions helped refine tools, clarify procedures and adapt intervention components to patients' realities. Dialogue between experiential and professional perspectives was described as mutually beneficial and progressively strengthened trust within the team: *What was nice over time was that there was more trust, but also a better understanding of what each person could bring. (PP03)*.

PPs also reported that participatory processes fostered team cohesion and shared understanding, particularly through co‐construction workshops, which facilitated collaboration across professional and experiential boundaries: *I hope that through these co‐construction workshops, we managed to create some cohesion within the teams, which helped facilitate connections between people. (PP01)*.

At an individual level, engagement supported the development of social and professional networks, extending beyond the PROOF project. Opportunities to connect with other PPs, researchers and healthcare professionals, as well as to attend scientific events, were seen as particularly valuable: *It allowed me to build a network, and that's extremely important. It also allowed me to attend the conference in Lyon [International conference on patient partnership in healthcare], which was always a valuable opportunity for exchanges. (PP05)*.

Finally, participants described gains in skills, confidence and reflexivity related to participatory research and peer support implementation. For some, PROOF constituted a reference experience that could be mobilised in future projects: *Everything I did in the PROOF programme became a kind of stepping stone. It's really a reference project for me. (PP03)*.

## 4 Discussion

This study explored PPs' experiences of participatory governance within the PROOF programme, a multicentre PAR initiative aimed at co‐designing and implementing a peer support intervention in oncology [24, 25]. By foregrounding PPs' perspectives and examining the concrete arrangements through which participation was organised, this study contributes to the growing literature on how participatory governance is enacted in practice, beyond normative calls for PPI.

Overall, PPs described their involvement as meaningful, evolving and sustained over time. Their accounts highlight that participatory governance in PROOF was not limited to symbolic inclusion, but relied on deliberate organisational choices that shaped relationships, roles and decision‐making processes. At the same time, persistent tensions related to administrative procedures, research timelines and institutional constraints illustrate the fragility of participatory ambitions when confronted with existing research infrastructures.

Interpreted through the BEE research framework, these findings offer insights into the relational and structural conditions required to support equitable patient engagement in complex oncology research programmes [31].

### 4.1 From Recruitment to Engagement: Recognition and Bidirectionality as Enabling Mechanisms

A key finding of this study concerns the role of early recognition in initiating and sustaining patient partner engagement. PPs consistently described being personally invited by healthcare professionals or researchers as a decisive trigger for their involvement. This mode of recruitment functioned as a strong signal of trust and legitimacy, positioning experiential knowledge as valuable from the outset. Involving PPs in the initial research stages may improve patients' comfort during data collection [14, 32, 33].

Within the BEE framework, such early recognition can be understood as a prerequisite for bidirectionality [31]. Rather than positioning patients as passive contributors to predefined agendas, personal invitations opened a space for reciprocal engagement in which patients perceived themselves as legitimate partners. Importantly, bidirectionality was not confined to initial recruitment but was progressively reinforced through patients' integration into decision‐making arenas such as steering committees and co‐construction workshops. PPs reported that their experiential knowledge was not only solicited but actively mobilised to shape tools, procedures and intervention components, illustrating a shift from consultation towards co‐production [8, 21].

However, our findings nuance idealised representations of co‐production by highlighting its temporal and relational dimensions [34]. Bidirectional engagement did not emerge spontaneously but developed through repeated interactions, clear role definition and ongoing trust‐building. This observation resonates with PAR literature, which emphasises that equitable partnerships are the result of sustained relational work rather than formal structures alone. It also suggests that early phases of participatory projects play a critical role in setting the conditions for later collaboration.

### 4.2 Equity Through Intentional Governance Structures and Supportive Arrangements

Equity, as conceptualised within the BEE framework, extends beyond interpersonal respect to encompass the organisational conditions that enable sustained and meaningful participation. In PROOF, equity was actively supported through the combination of multiple governance spaces, including committees specifically dedicated to PPs. These spaces enabled free expression, peer exchange and the development of a shared identity among PPs, particularly in a multicentre context characterised by heterogeneous profiles and settings.

Beyond committee structures, relational and material supports such as individual onboarding meetings, continuous availability of the coordinating team, and financial compensation also played a crucial role in sustaining engagement. These measures contributed to clarifying expectations, addressing emerging difficulties and recognising PPs' time and expertise [35]. Together, they illustrate how equity is enacted through a combination of governance design, relational practices and concrete resources.

**Figure 2:**
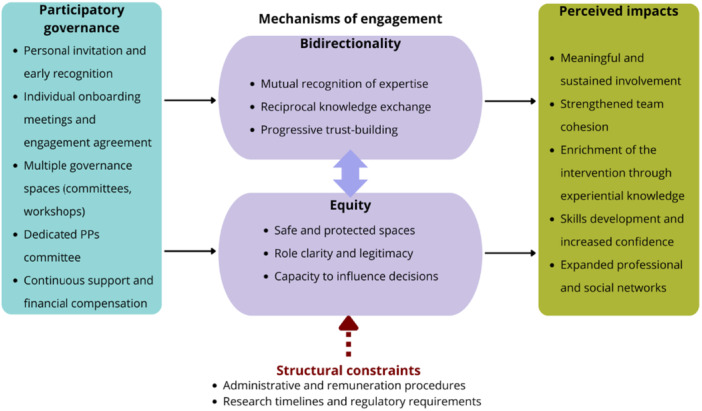
Mechanisms linking participatory governance to patient partners' experiences in the PROOF programme. This figure is informed by the bidirectional engagement and equity (BEE) research framework, which conceptualises equitable patient engagement through the dual mechanisms of bidirectionality and equity [31]. The model was used as an interpretative lens to organise the relationships between participatory governance arrangements, engagement mechanisms, and perceived impacts.

Nevertheless, PPs' experiences also reveal the limits of equity within existing institutional frameworks. Administrative and financial constraints, particularly regarding remuneration procedures, were repeatedly identified as sources of frustration and uncertainty. These challenges echo broader critiques in the PPI literature, which point to a persistent misalignment between participatory aspirations and regulatory or administrative systems [18, 19, 36]. While part of these constraints reflects structural conditions that exceed the scope of any single project in France, the absence of a harmonised national framework for compensating PPs requires each institution to adapt generic administrative procedures to heterogeneous individual statuses, our experience also points to concrete, project‐level lessons. In PROOF, the diversity of PPs' employment and social statuses meant that each compensation file required individual administrative processing, and payment timelines could not always be anticipated or communicated to PPs in advance, which amplified frustration. In retrospect, the administrative dimension of patient partnership, particularly the anticipation of compensation circuits, deserved as much early attention as the scientific dimension of the project, a balance that proved difficult to strike in practice. Future projects could mitigate these difficulties by: (i) identifying each patient partner's employment and social status at the very start of their engagement and mapping out the corresponding payment circuit at that point, rather than once activities have already taken place; (ii) budgeting compensation as a dedicated, ring‐fenced line at the grant application stage; (iii) providing PPs, from the outset, with transparent written information on payment modalities and realistic timelines; and (iv) designating an identified administrative referent for PPs compensation. Acknowledging these actionable levers does not negate the structural nature of the underlying constraints, but reframes their management as a shared responsibility between research teams and institutions (Figure 2).

### 4.3 Strengths and Limitations

This study has several strengths. It provides an in‐depth qualitative analysis of PPs' experiences within a large, multicentre public health research‐action programme and foregrounds patients' voices in the examination of participatory governance. The use of an independent interviewer and the involvement of PPs in reviewing analytical outputs further enhance the credibility and reflexivity of the study.

Nevertheless, some limitations should be acknowledged. This study focused exclusively on PPs' experiences during the first phase of the PROOF programme. As such, it does not capture the perspectives of other stakeholders, including researchers and healthcare professionals, nor does it reflect how perceptions of participatory governance may evolve over the full 4‐year duration of the project. Future studies should adopt a longitudinal and multi‐perspective approach to examine changes in experiences and perceptions of patient involvement over time and across stakeholder groups. Second, the findings are rooted in a PAR context, which is particularly conducive to intensive and sustained patient involvement. This may limit the direct generalisation of results to other research contexts, such as more conventional clinical or biomedical studies, where opportunities for patient partnership may be more constrained. However, rather than aiming for generalisability, this study seeks analytical and theoretical transferability by identifying mechanisms (recognition, bidirectionality, equity, organisational support) that may inform the design of participatory governance in other types of studies. Third, the interviewed sample was almost exclusively female (six of seven participants). This imbalance reflects two converging factors rather than a differential acceptance of the interview invitation. First, it partly mirrors the case‐mix of the participating units: several specialise in breast and gynaecological oncology, where the eligible patient population is predominantly female, although other units recruited from cancer sites affecting both women and men. Second, it is consistent with the well‐documented over‐representation of women among patient and public contributors in health research more broadly [37]. Nevertheless, the experiences, expectations and barriers reported here may not fully capture those of male PPs, and gendered dimensions of engagement (e.g., availability, caregiving responsibilities, communication preferences) could not be explored in this sample. Finally, all PPs who took part in Phase 1 of the PROOF programme had accepted the invitation to join and remained involved throughout, indicating full uptake and no attrition once the project was under way (one patient partner who had initially agreed withdrew before the effective start, for reasons related to her concurrent peer‐support role rather than to the research partnership, and was replaced). This high acceptance is consistent with the emphasis our results place on direct, personal invitation as an engagement lever, but may also reflect a self‐selection of patients already favourably disposed towards partnership, which should be borne in mind when interpreting their largely positive accounts.

### 4.4 Implications for Participatory Research in Oncology

The findings of this study have several implications for the design and conduct of participatory research in oncology. First, they underscore the importance of intentional and structured participatory governance. Meaningful patient partner involvement did not emerge spontaneously but was supported by clearly identified roles, multiple entry points into decision‐making, and dedicated spaces that recognised the specific needs of PPs.

Second, PPs' accounts suggest that recognition of experiential expertise must be continuously enacted through everyday practices, rather than assumed once partnership is formally established. Concrete mechanisms such as explicit invitations to contribute during meetings, facilitation styles that allow time and silences, and visible uptake of patient input, were described as key to building confidence and enabling PPs to feel like full members of the project.

The findings underline the added value of dedicated patient partner spaces. The PPs committee provided a protected environment for peer exchange, questions and shared learning, helping reduce feelings of intimidation and strengthening confidence and legitimacy over time. This suggests that dedicated spaces are not peripheral but constitute a core mechanism supporting sustained engagement.

Finally, the results point to the need for greater institutional alignment between participatory ambitions and existing regulatory and administrative frameworks. Patient engagement cannot be sustained solely through individual motivation; it relies on reciprocal engagement from all actors involved in the research process, including researchers, healthcare professionals, and institutional partners. In PROOF, PPs' involvement was supported when non‐research stakeholders actively adapted their practices by facilitating participation in meetings, remaining available for individual support, and recognising experiential knowledge as a legitimate contribution.

Persistent challenges related to remuneration and research timelines risk undermining equity and long‐term involvement. Addressing these issues will require not only project‐level anticipation but also broader policy‐level guidance and national recommendations to support sustainable patient partnership in oncology research. In this respect, international infrastructures such as INVOLVE, SPOR and PCORI have produced practical guidance (e.g., on payment/recognition, budgeting, and role clarification) that can help operationalise and standardise equitable engagement whereas, in France, the relative absence of harmonised national guidance leaves research teams to ‘reinvent’ procedures locally, often at the cost of delays and unequal treatment. However, even where compensation guidance exists, implementation often remains difficult in practice because of budget constraints and, critically, the absence of institutional policies and payment procedures that would allow research teams to operationalise compensation consistently across diverse patient‐partner statuses [19].

## 5 Conclusion

Patient partner involvement in PAR in oncology is feasible and adds substantial value when supported by structured governance and dedicated spaces for engagement. The main challenges identified relate not to patients' capacity to contribute, but to administrative and procedural constraints. Addressing these issues through anticipatory planning and clearer regulatory frameworks is essential to sustain meaningful patient partnership in research.

## Author Contributions

**Yaël Busnel:** formal analysis, conceptualisation, writing – original draft, methodology, validation, writing – review and editing. **Aline Rollet:** investigation, writing – original draft, writing – review and editing, formal analysis. **Aline Cristiani:** validation, formal analysis, writing – original draft, writing – review and editing. **Véronique Rivoire:** writing – original draft, writing – review and editing, validation, formal analysis. **Laurie Panse:** validation, formal analysis, writing – review and editing, writing – original draft. **Julie Haesebaert:** conceptualisation, funding acquisition, writing – original draft, writing – review and editing, supervision, methodology, validation. **Kepenekian Vahan:** supervision, resources. **Dalle Stéphane:** supervision, resources. **Sébastien Couraud:** supervision, resources. **Mellie Heinmann:** supervision, resources. **Hallouche Nabil:** supervision, resources. **Tournigand Christophe:** supervision, resources. **Wassermann Johanna:** supervision, resources. **Boissel Nicolas:** supervision, resources. **Rabian Florence:** supervision, resources. **Mebarki Soraya:** supervision, resources. **Pauline Maisani:** supervision, resources. **Aurélien Troisoeufs:** supervision, resources. **Anne Termoz:** supervision, resources. **Pascale Sontag:** supervision, resources. **Magalie Girodet:** supervision, resources. **Mathilde Lochmann:** validation, resources. **Véronique Christophe:** supervision, resources. **Stéphane Cognon:** validation, formal analysis. **Sarah Prudhomme:** validation, formal analysis. **Claude Ganter:** validation, formal analysis. **Alice Gros:** validation, formal analysis. **Alexandra Villatte:** validation, formal analysis.

## PROOF Investigators Group

We thank the PROOF investigators group: Dr Kepenekian Vahan, Pr Dalle Stéphane, Pr Sébastien Couraud from Hospices Civils de Lyon; Dr Mellie Heinmann from Centre Léon Bérard; Pr Hallouche Nabil from GHU Paris Psychiatrie &; neurosciences and Pr Tournigand Christophe; Dr Wassermann Johanna, Pr Boissel Nicoal, Dr Rabian Florence, Dr Mebarki Soraya from Assistance Publique – Hôpitaux de Paris; Pauline Maisani from Assistance Publique – Hôpitaux de Paris; Aurélien Troisoeufs from GHU Paris Psychiatrie and Neurosciences; Anne Termoz from Hospices Civils de Lyon; Pascale Sontag, Magalie Girodet, Mathilde Lochmann and Véronique Christophe from Centre Léon Bérard. This research was supported by the French National Cancer Institute (INCa). The funding body played no role in the design of the study, in the collection, analysis and interpretation of data, nor in drafting the manuscript.

## Ethics Statement

The study received ethical approval by the Medical Ethical Review Board of the Hospices Civils de Lyon (2024‐01‐25‐04).

## Consent

The authors have nothing to report.

## Conflicts of Interest

The authors declare no conflicts of interests.

## Supporting information


**Additional Files 1:** Overview of the the PaRole OncO France (PROOF) programme.

**Additional Files 2:** Interview guide for patient partners.

## Data Availability

The data that support the findings of this study are available from the corresponding author upon reasonable request.
