# Supplementary figures and images for: Patients as Co‐Researchers in Oncology: A Qualitative Exploration of Participatory Governance
Source: Health Expect. 2026 Jul 9;29(4):e70762. doi: 10.1111/hex.70762 (PMC13347621; doi:10.1111/hex.70762)

**Additional files 1. Overview of the the PaRole OncO France (PROOF) programme**

**
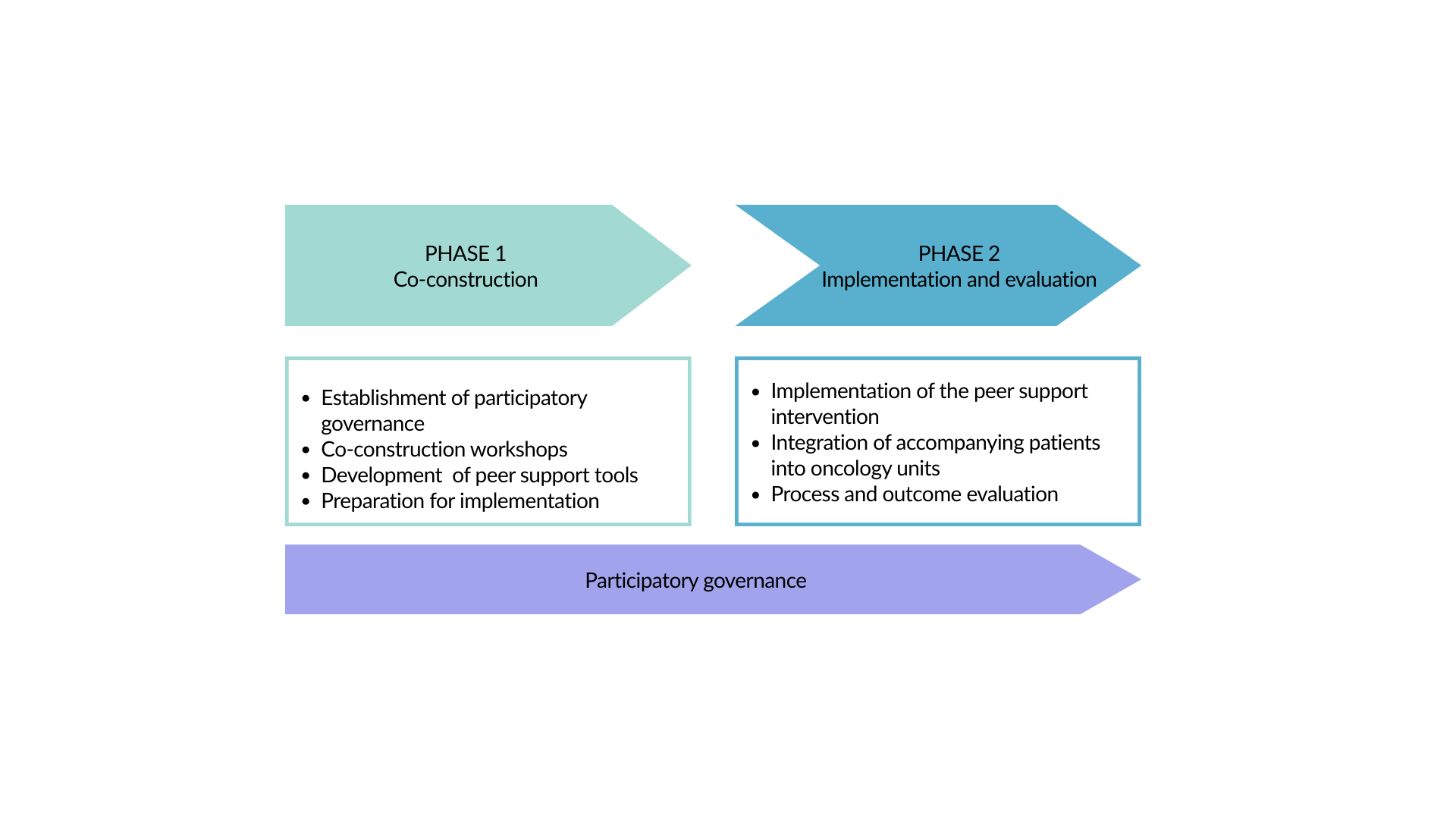
**

Supplement: Supplementary file 1 — Additional Files 1: Overview of the the PaRole OncO France (PROOF) programme. [file HEX-29-e70762-s001.docx]
