# Supplementary material for: Patients as Co‐Researchers in Oncology: A Qualitative Exploration of Participatory Governance
Source: Health Expect. 2026 Jul 9;29(4):e70762. doi: 10.1111/hex.70762 (PMC13347621; doi:10.1111/hex.70762)
Supplement: Supplementary file 2 — Additional Files 2: Interview guide for patient partners. [file HEX-29-e70762-s002.docx]

**Interview guide for patient partners**

PREAMBLE

Information and consent form

- Ensure that the participant present has signed the ICF.
- Ensure that the participant agrees to the interview being recorded.

Context

- Thank the participant for agreeing to answer our questions as part of our study.
- Specify that the interview will last approximately 60 minutes.
- Context

Interview rules

- Everything that is said will remain confidential. To protect your privacy, your name will not appear anywhere and you will remain completely anonymous.
- At any time during our conversation, please let me know if you have any questions or if you would prefer not to answer a particular question. You may also decide to stop the interview at any time.
- Remember that we want to know what you think and how you feel. There are no right or wrong answers.
- Ensure that the participant agrees to the interview being recorded.

[ START RECORDING]

1. Tell me how you heard about the study.
   1. What was your first impression?
   2. What motivated you to participate? What were your fears and expectations?
2. Tell me about your participation in the study.
   1. Can you tell me how your participation in the various commitees went?
   2. Did you notice any changes in your role over time?
3. How did you feel as a patient partner? And as a group?
   1. How involved did you feel in the discussions and decision-making?
   2. How would you describe your relationship with the project team?
4. In your opinion, what was the impact of your participation?
   1. What did you learn or gain from it, either personally or collectively?
   2. Did you feel that your participation was valuable to the project and the professionals involved?
5. Did you encounter any factors that facilitated or hindered your participation?
   1. In terms of organization (meetings, communication, etc.), what was helpful or limiting?
   2. In terms of human relations (relationships with professionals, other patient partners, etc.), what was helpful or limiting?
6. What advice would you give to a future patient partner joining a project?

Closing: Are there any other points you would like to discuss?

Thank you very much for participating in this interview.

[End recording]
